# Coevolved Multidrug-Resistant HIV-1 Protease and Reverse Transcriptase Influences Integrase Drug Susceptibility and Replication Fitness

**DOI:** 10.3390/pathogens10091070

**Published:** 2021-08-24

**Authors:** Supang A. Martin, Patricia A. Cane, Deenan Pillay, Jean L. Mbisa

**Affiliations:** 1 Antiviral Unit, Virus Reference Department, Public Health England, London NW9 5EQ, UK; supang.martin@gmail.com (S.A.M.); pat.cane@phe.gov.uk (P.A.C.); 2 Division of Infection and Immunity, University College London, London WC1E 6BT, UK; d.pillay@ucl.ac.uk

**Keywords:** HIV-1, single genome sequencing, drug resistance, integrase strand transfer inhibitors, replication fitness

## Abstract

Integrase strand transfer inhibitors (InSTIs) are recommended agents in first-line combination antiretroviral therapy (cART). We examined the evolution of drug resistance mutations throughout HIV-1 *pol* and the effects on InSTI susceptibility and viral fitness. We performed single-genome sequencing of full-length HIV-1 *pol* in a highly treatment-experienced patient, and determined drug susceptibility of patient-derived HIV-1 genomes using a phenotypic assay encompassing full-length *pol* gene. We show the genetic linkage of multiple InSTI-resistant haplotypes containing major resistance mutations at Y143, Q148 and N155 to protease inhibitor (PI) and reverse transcriptase inhibitor (RTI) resistance mutations. Phenotypic analysis of viruses expressing patient-derived IN genes with eight different InSTI-resistant haplotypes alone or in combination with coevolved protease (PR) and RT genes exhibited similar levels of InSTI susceptibility, except for three haplotypes that showed up to 3-fold increases in InSTI susceptibility (*p* ≤ 0.032). The replicative fitness of most viruses expressing patient-derived IN only significantly decreased, ranging from 8% to 56% (*p* ≤ 0.01). Interestingly, the addition of coevolved PR + RT significantly increased the replicative fitness of some haplotypes by up to 73% (*p* ≤ 0.024). Coevolved PR + RT contributes to the susceptibility and viral fitness of patient-derived IN viruses. Maintaining patients on failing cART promotes the selection of fitter resistant strains, and thereby limits future therapy options.

## 1. Introduction

Since the introduction of combination antiretroviral therapy (cART) against HIV-1 in the late 1990s, the number of overall new HIV infections and AIDS-related deaths has decreased [1]. Despite this, the development of resistance to antiretrovirals (ARVs) remains a barrier to the treatment of HIV infection, especially in low- and middle-income countries, and work is ongoing to produce drugs that target novel steps of the viral life cycle. One of the later classes of drugs to be approved for clinical use targets the integration of the virus into the host cell genome, specifically the DNA strand transfer reaction [2,3]. To date, there are five licensed integrase strand transfer inhibitors (INSTIs), raltegravir (RAL), elvitegravir (EVG), dolutegravir (DTG), bictegravir (BIC) and cabotegravir (CAB), that are available singly or as combination pills with RTIs [4].

Originally, RAL was used as part of a second-line treatment or salvage regimen for those failing previous lines of therapy [5]. However, InSTIs are now recommended for use as a third agent in a nucleos(t)ide reverse transcriptase inhibitor (NRTI)-based first-line therapy [6,7]. Thus, it is highly likely that patients going onto InSTI-containing treatment will develop alongside or harbour viruses containing protease inhibitor (PI) and RTI resistance mutations. However, little is known about the full effects and coevolution of drug resistance in the full-length HIV-1 *pol* gene, the target of the four main classes of ARVs used in cART: PIs, NRTIs, non-nucleoside reverse transcriptase inhibitors (NNRTIs) and InSTIs. This is because routine genotypic resistance testing in HIV-infected patients normally involves standard “bulk” population sequencing of viral genes of interest; usually protease (PR) and the N terminal of RT, or the catalytic core domain of integrase (IN) separately, or, alternatively, short read (75 to 300 bp) next-generation sequencing is used [8,9]. In addition, most phenotypic drug susceptibility assays have been developed to study the drug susceptibility of smaller patient-derived *gag-pol* fragments [10,11,12,13]. A limited number of studies have investigated the drug susceptibility of patient-derived full-length *pol* genes [14,15,16]. Consequently, not much is known about the effects of different combinations of PI, RTI and InSTI resistance mutations on overall drug susceptibility and viral fitness. These data may be important in informing how best to use drugs targeting all three genes in clinical practice. Here, we use a full-length *pol* single-genome sequencing assay to investigate the development, evolution and linkage of PI, RTI and InSTI resistance mutations in a patient undergoing InSTI-containing therapy. We also investigate the effect on InSTI susceptibility and viral fitness of patient-derived *IN* only or in combination with coevolved *PR* and *RT*, using a single-replication-cycle drug susceptibility assay. 

## 2. Results

### 2.1. Single-Genome Sequencing of Full-Length HIV-1 pol Gene in Longitudinal Samples from a Patient Failing RAL-Containing Therapy

Single genomes were generated from five out of six samples over a 16-month period (Table 1). A total of 117 single genomes were generated at the following time points: before initiation of RAL therapy (preRAL; n = 16), 4 and 5 months after initiation of RAL therapy (4RAL and 5RAL; n = 26 and 23, respectively), 4 months after cessation of RAL therapy (4post; n = 39) and 2 weeks after re-initiation of RAL therapy (reRAL; n = 13).

**Figure 1:**
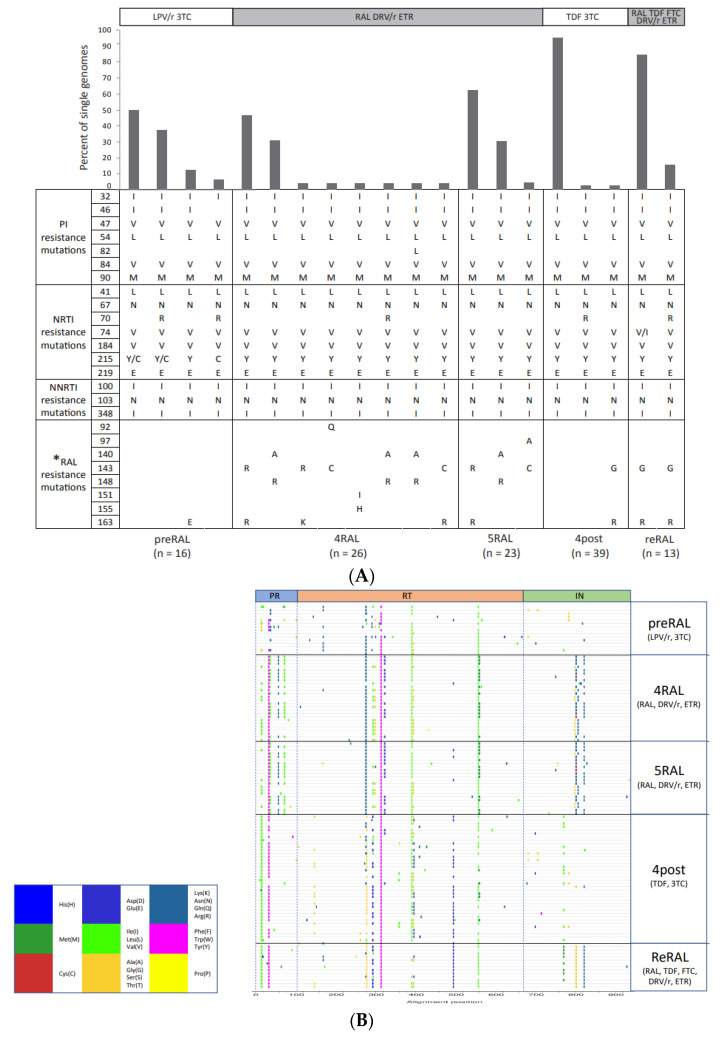
Genetic linkage of drug resistance mutations in PR, RT and IN genes from patient failing RAL-containing therapy. (**A**) Genetically linked major PI, NRTI, NNRTI and RAL resistance mutations identified by single genome sequencing in longitudinal samples are shown in columns. The percent of single genomes containing the linked resistance mutations at each time point are shown in bar graphs above the mutation columns. The details of treatment are indicated above the bar graphs and the number of single genomes generated at each time point is indicated below each figure. The bar graph on the right-hand side shows the frequency of the mutations in the Stanford HIV Drug Resistance Database in treatment-experienced patients infected with subtype B. (**B**) Highlighter plot of amino acid differences throughout the pol gene of the patient single genomes during the different time points and treatment regimens. The differences are in reference to the ancestral single genome at the preRAL time point. preRAL = before initiation of RAL therapy; 4RAL, 5RAL and 8RAL = 4, 5 and 8 months after initiation of RAL therapy, respectively; 1post, 3post and 4post = 1, 3 and 4 months after stopping RAL therapy; reRAL = 0.5 months after reinitiating RAL therapy; * = accessory RAL resistance mutations included; ** = L74V only; *** = T215Y only; **** = Y143R and Y143C only.

Analysis of the 16 single genomes before exposure to RAL (preRAL) revealed no RAL resistance mutations, except for the presence of an amino acid substitution (G163E) in a single genome at a position associated with RAL resistance (Figure 1). On the other hand, all single genomes generated after initiation of RAL treatment (4RAL and 5RAL; n = 49) contained RAL resistance mutations at all the three major RAL resistance positions at 4RAL, with these being: Y143R/C (n = 15), Q148R (n = 10) and N155H (n = 1). The major RAL resistance pathways were reduced to two at 5RAL, with these being: Y143R/C (n = 16) and Q148R (n = 7). None of the three major RAL resistance-associated mutations at positions 143, 148 and 155 observed at 4RAL were found on the same genome. However, all three major RAL resistance mutations were found to be linked to one of the following accessory mutations: E92Q, T97A, G140A, V151I and G163R/K. Analysis of the genetic linkage of the major and accessory resistance mutations revealed seven different RAL-associated haplotypes in the 49 single genomes during the first round of RAL treatment (4RAL and 5RAL), with these being: Y143R + G163R (n = 27), Y143R + G163K (n = 1), Y143C + E92Q (n = 1), Y143C + G163R (n = 1), Y143C + T97A (n = 1), Q148R + G140A (17) and N155H + V151I (n = 1). Six of the haplotypes were present at 4RAL, and this number decreased to three a month later at 5RAL. However, at both time points, two haplotypes, Y143R + G163R and Q148R + G140A, dominated the population constituting 46.2% and 38.5% of the population at 4RAL and 65.2% and 30.4% at 5RAL, respectively.

Within four months following the withdrawal of RAL treatment (4 post), 97.4% (38/39) of the single genomes contained no RAL resistance-associated mutations, with only one single genome still harbouring the G163R mutation and a novel substitution at major resistance position 143 (Y to G). This minor variant was not detected in any of the single genomes at 4RAL or 5RAL. Two weeks after RAL therapy was re-initiated (ReRAL), this novel Y143G + G163R mutant dominated the viral population with all of the single genomes (n = 13) containing the double mutation.

### 2.2. The Development and Linkage of Drug Resistance Mutations in Full-Length HIV-1 pol Gene

We investigated the linkage of RAL resistance mutations in IN to drug resistance mutations in PR and RT. We found little variation in the composition of PI and RTI resistance mutations over the sampling period. All 117 amplified single genomes contained numerous PI (V32I, I47V, I54L, I84V, and L90M), NRTI (M41L/I, D67N, L74V/I, M184V, T215Y/C and K219E) and NNRTI (L100I, K103N and N348I) resistance mutations (Figure 1). This is consistent with the extensive ART experience of the patient.

These PI and RTI resistance mutations were maintained in the viral population, even in the absence of the respective drugs. For example, the PI resistance mutations in the preRAL time point were still present at the 4post time point when the patient was no longer on PIs (Figure 1). We observed the genetic linkage of drug resistance mutations across the full-length *pol* gene in 100% (n = 64) of single genomes that contained RAL resistance mutations (Figure 1A). Analysis of the Stanford HIV Drug Resistance Database showed that these mutations were identified in a significant proportion of patient samples infected with subtype B, except for the PI V82L, NRTI L74I and T215C, and InSTI Y143G (Figure 1A). However, the frequency of linkage of the mutations could not be verified, as the data were from population-based Sanger sequencing, and not single-genome sequencing.

Although the major PI and RTI resistance mutations were maintained throughout the study period, regardless of treatment regimen, there was selection for or against the major RAL resistance mutations and other mutations in all three gene regions, depending on the treatment regimen (Figure 1B). 

### 2.3. Intrapatient Evolution of InSTi Susceptibility

**Figure 2:**
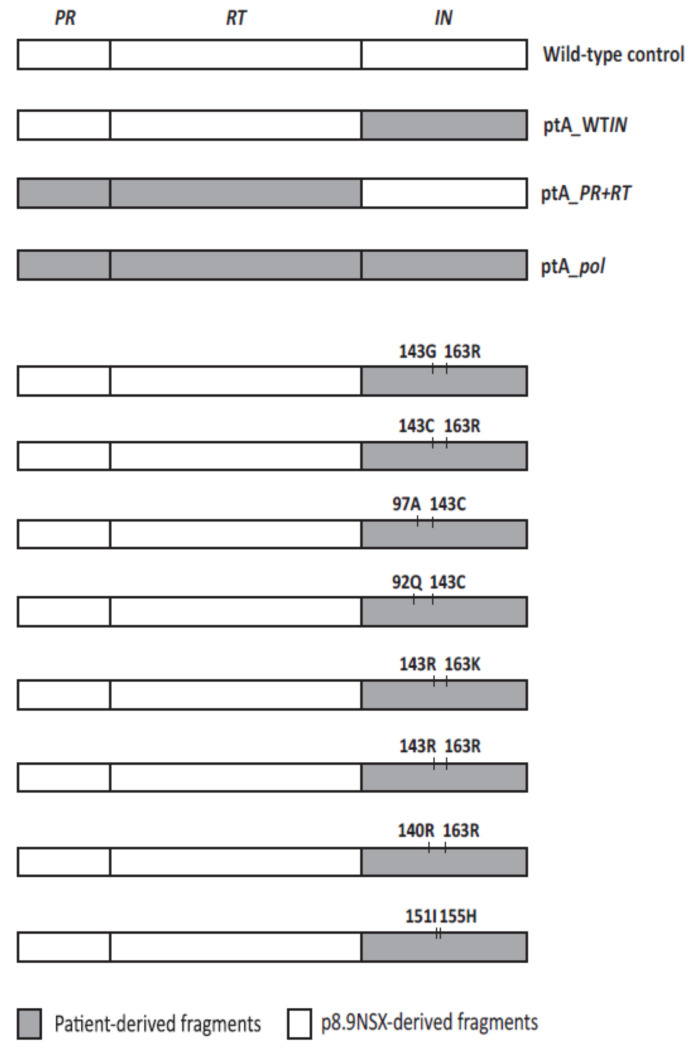
A schematic representation of recombinant viral vectors used in phenotypic drug susceptibility and viral fitness assays. Patient-derived *IN* genes containing different RAL-resistant haplotypes or wild-type only were sub-cloned into p8.9NSX vector. Patient-derived full-length *pol* vectors were generated by subcloning the patient-derived *IN* genes into a vector expressing the PR + RT fragment from the patient. The patient-derived *PR* + *RT* fragment contained the PI resistance mutations L10F, V32I, I47V, I54L, A71T, I84V and L90M, and RTI resistance mutations M41L, D67N, I74V, M184V, T215Y, K219E, L100I, K103N and N348I. Wild-type control = p8.9NSX wild-type subtype B; ptA_WT*IN* = patient-derived wild-type in *IN*; ptA_*PR* + *RT* = patient-derived *PR* and *RT*; ptA_*pol* = patient-derived full-length *pol*, wild-type in *IN*. Vectors with patient-derived full-length *pol* containing RAL-resistant haplotypes are not shown.

**Figure 3:**
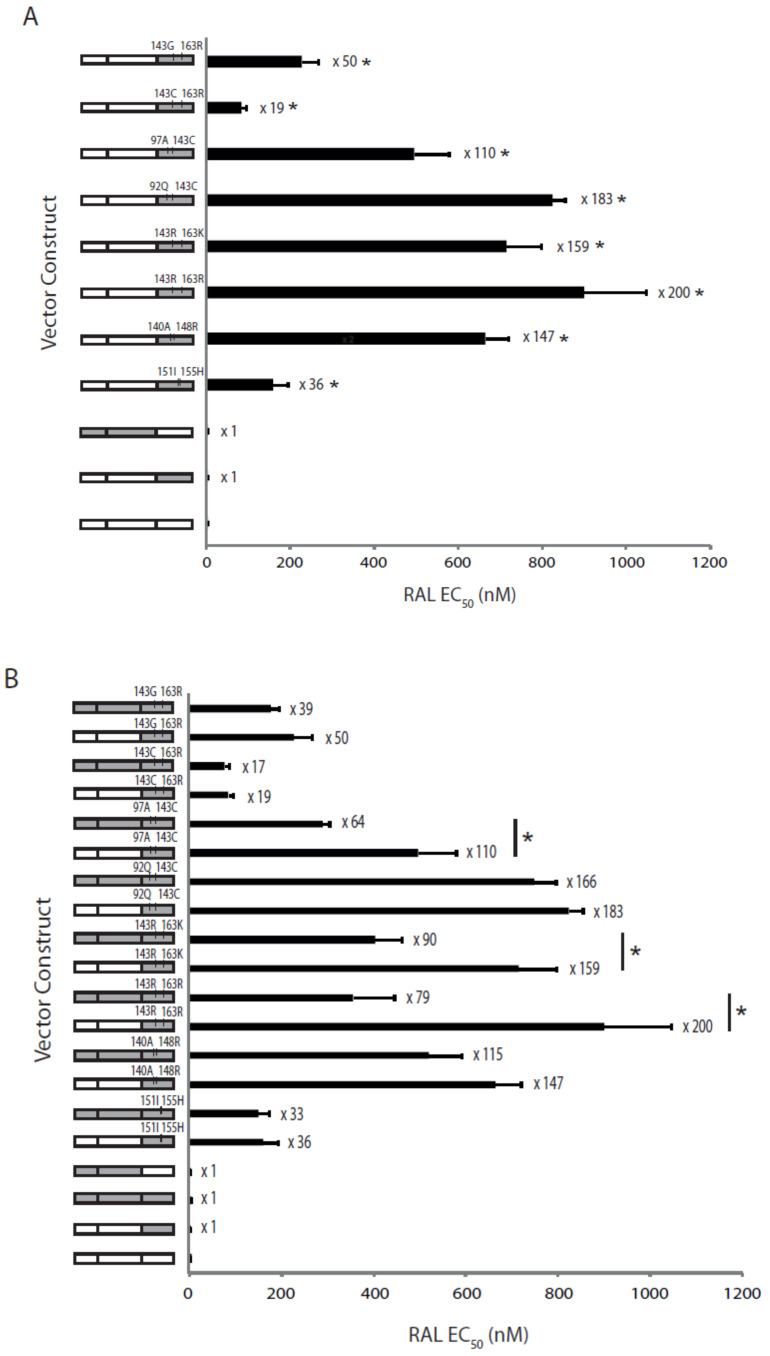
Susceptibility to RAL exhibited by recombinant viruses expressing patient-derived HIV-1 gene fragments. (**A**) Susceptibility to RAL exhibited by recombinant viruses expressing patient-derived *IN* genes only. (**B**) Comparison of RAL susceptibilities exhibited by recombinant viruses expressing patient-derived *IN* genes only or full-length *pol* genes. Error bars represent standard error of the mean of 6 to 12 independent experiments. Fold change in EC_50_ values compared to the p8.9NSX wild-type control are indicated next to each bar. Viruses exhibiting a significantly higher RAL EC_50_ (*p* < 0.05) compared to wild-type control (**A**) or their full-length *pol* counterpart (**B**) are indicated with *.

We investigated the effect of the different drug resistance-associated mutations identified during the development of RAL resistance on RAL susceptibility. We generated nine recombinant virus vectors expressing patient-derived *IN* genes with eight different RAL-resistant haplotypes and a wild-type sequence that were identified by single genome sequencing (Figure 2). As expected, the RAL EC_50_ of the virus expressing the patient-derived wild-type *IN* from the pre-RAL time-point (ptA_WT*IN*) was equivalent to that of the wild-type control virus (p8.9NSX) at 4.2 ± 0.11 vs. 4.5 ± 0.37 nM (*p* = 0.76; Figure 3A). In contrast, all viruses containing patient-derived *IN* from single genomes sampled during RAL treatment exhibited significant decreases in RAL susceptibility of up to 200-fold. compared to that of the wild-type *IN* control with EC_50_ values ranging from 84.6 ± 10.5 to 900 ± 145.2 nM (*p* ≤ 0.0001). Interestingly, the major RAL-resistant haplotype at 4RAL and 5RAL, Y143R + G163R, had the highest decrease in RAL susceptibility (200-fold), compared to a moderate decrease in RAL susceptibility of 50-fold for the Y143G + G163R haplotype, that emerged during re-initiation of RAL therapy.

**Figure 4:**
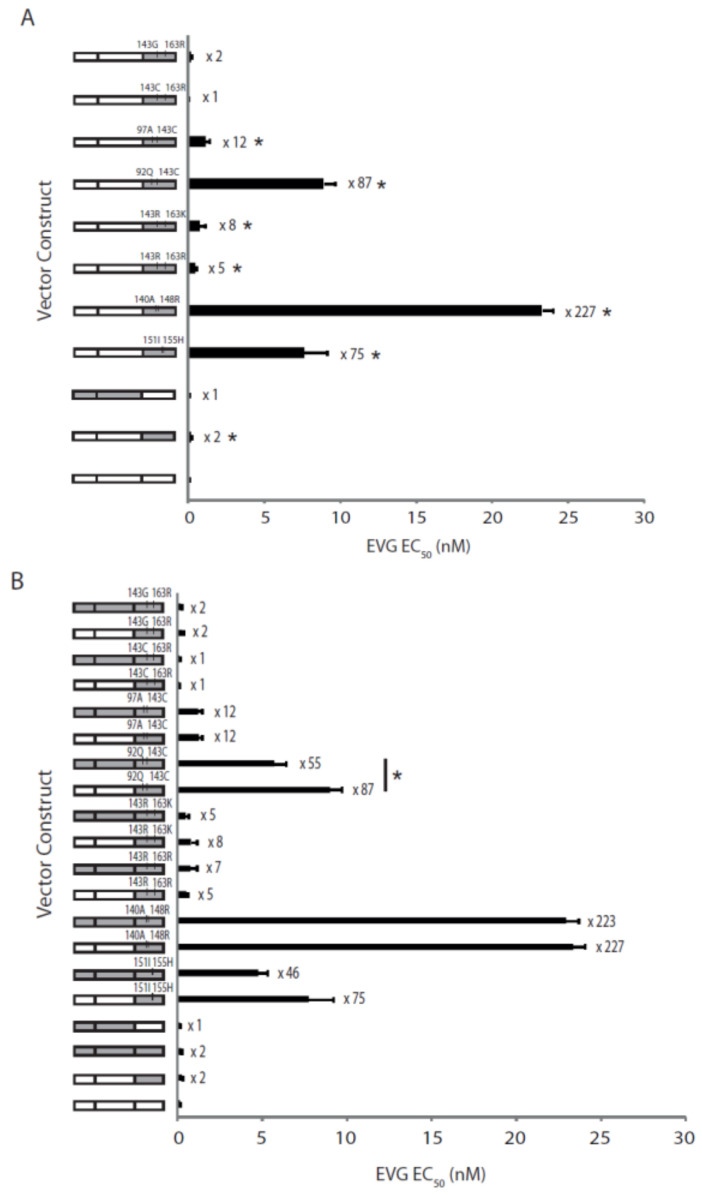
Cross-resistance to EVG exhibited by recombinant viruses expressing patient-derived HIV-1 gene fragments. (**A**) Susceptibility to EVG exhibited by recombinant viruses expressing patient-derived *IN* genes only. (**B**) Comparison of EVG susceptibilities exhibited by recombinant viruses expressing patient-derived *IN* genes only or full-length *pol* genes. Error bars represent standard error of the mean of 6 to 12 independent experiments. Fold change in EC_50_ values, compared to the p8.9NSX wild-type control are indicated next to each bar. Viruses exhibiting a significantly higher EVG EC_50_ (*p* < 0.05) compared to wild-type control (**A**) or their full-length *pol* counterpart (**B**) are indicated with *.

Next, we investigated the susceptibility of the viruses expressing patient-derived fragments against a different InSTI, EVG. Interestingly, the EVG EC_50_ of the virus expressing the patient-derived wild-type *IN* from pre-RAL time point (ptA_WT*IN*) was significantly higher compared to the wild-type control (p8.9NSX) at 0.24 ± 0.026 vs. 0.1 ± 0.016 nM (*p* = 0.0002; Figure 4A). This is consistent with other studies, that have shown a 2- to 3-fold increase in EVG EC_50_ for viruses expressing *IN* genes from InSTI-naïve patients compared to wild-type controls [17,18]. Two viruses expressing patient-derived *IN* containing the RAL resistance mutations, Y143C + G163R and Y143G + G163R, also exhibited EVG EC_50_ values similar to that of the wild-type control at 0.06 ± 0.022 and 0.22 ± 0.088 nM, respectively (*p* ≥ 0.12). The remaining six viruses expressing patient-derived *IN* only were found to have significantly higher EVG EC_50_ values compared to the wild-type control, ranging from 0.48 ± 0.064 to 23.31 ± 0.69 nM (*p* < 0.05). This includes the virus expressing the Y143R + G163R mutation combination, which exhibited the highest fold change in RAL EC_50_ (200-fold), but which only resulted in a 5-fold change in EVG susceptibility. The highest fold change in EVG susceptibility (227-fold) was exhibited by the virus expressing the Q148R + G140A mutation combination, which also resulted in a very high fold change in RAL susceptibility (147-fold).

### 2.4. The Effects of Coevolved PR and RT Genes on Susceptibility to InSTIs

To determine if the coevolved *PR* and *RT* genes influence the susceptibility to the InSTIs RAL and EVG, we generated recombinant vectors expressing patient-derived full-length *pol* gene. The virus expressing the patient-derived full-length *pol* with resistance mutations in *PR* and *RT* but wild-type in *IN* (designated ptA_*pol*), as well as the virus expressing the patient-derived *PR* and *RT* only (designated ptA_*PR* + *RT*) had RAL susceptibilities comparable to that of the wild-type control virus (p8.9NSX) with EC_50_ values of 5.5 ± 1.1 nM, 4.1 ± 0.48 and 4.5 ± 0.37 nM, respectively (*p* ≥ 0.30; Figure 3B). Overall, the viruses expressing patient-derived full-length *pol* from time points during RAL treatment exhibited RAL susceptibilities that were similar to viruses expressing respective patient-derived *IN* only, except for the Y143R + G163R, Y143R + G163K and Y143C + T97A mutation combinations. For these three viruses, the patient-derived *IN* only showed significantly greater decreases in RAL susceptibilities than the respective full-length *pol* (*p* ≤ 0.032). This suggests that the coevolved *PR* and *RT* can confer a negative effect on resistance to RAL, which is dependent on the combination of resistance mutations in *IN*.

To determine if the coevolved *PR* and *RT* also influences EVG susceptibility, we investigated the EVG susceptibilities of viruses expressing patient-derived full-length *pol* (Figure 4B). Similar to the virus expressing patient-derived wild-type *IN* gene only (ptA_WT*IN*), the virus expressing patient-derived full-length *pol* with resistant *PR* and *RT* genes but wild-type *IN* gene (ptA_*pol*) also exhibited a significantly higher EVG EC_50_ compared to wild-type control virus at 0.18 ± 0.033 vs. 0.1 ± 0.016 nM (*p* = 0.03). Overall, the EVG EC_50_ values of viruses expressing patient-derived full-length *pol* were similar to that of viruses expressing patient-derived *IN* gene only, with the exception of the virus expressing Y143C + E92Q mutations, which showed a significant decrease in EVG susceptibility for patient-derived *IN* gene only compared to full-length *pol* gene (*p* = 0.0064). Again, this may indicate that the patient coevolved *PR* and *RT* genes’ influence on the susceptibility to EVG is dependent on the combination of InSTI resistance mutations; however, this effect is different between EVG and RAL.

### 2.5. The Effects of Patient-Derived pol Gene Fragments on Viral Replicative Fitness

**Figure 5:**
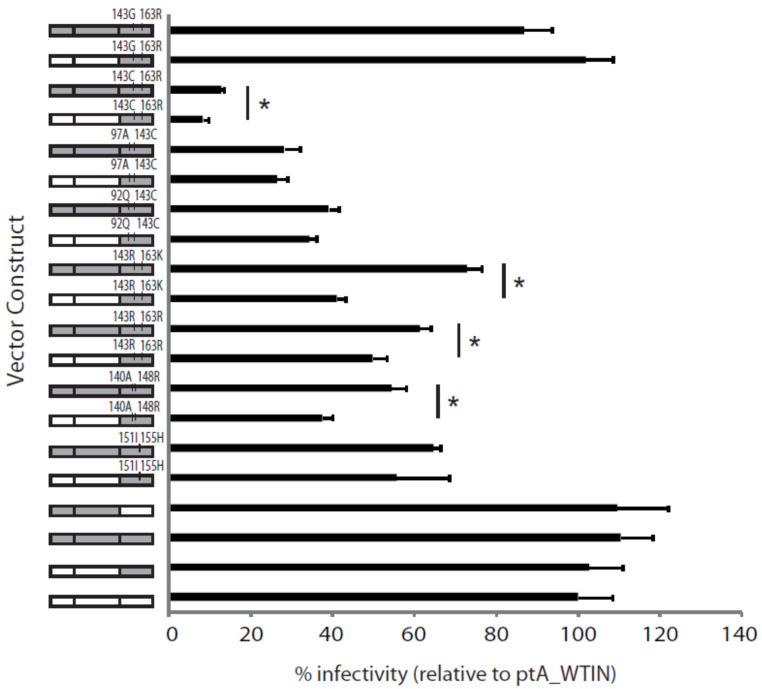
The effect on viral replicative fitness of patient-derived *IN* gene only or full-length *pol* gene. Viral infectivity in a single-replication-cycle assay was used to determine the replicative fitness of recombinant viruses expressing patient-derived *IN* gene only or full-length *pol* genes. The replicative fitness relative to ptA_WT*IN* control (vector containing patient-derived *IN* only from the pre-RAL time point), set at 100%, is shown for each virus. Significant differences (*p* < 0.05) between patient-derived *IN* only viruses and their full-length *pol* counterparts are indicated with *. The error bars represent standard error of the mean of six independent experiments.

Next, we tested the replicative fitness of the viruses expressing patient-derived full-length *pol* or *IN* gene only (Figure 5). Using a single-replication-cycle assay, the wild-type control virus, the virus expressing patient-derived *PR* + *RT* (ptA_*PR* + *RT*) and the virus expressing patient-derived full-length *pol* with wild-type *IN* showed similar replicative fitness to the virus expressing patient-derived wild-type *IN* only (ptA_WT*IN*; set to 100%) at 110.5 ± 7.7%, 109.7 ± 12.3% and 102.9 ± 8%, respectively (*p* ≥ 0.39). 

Interestingly, the only other virus that showed replicative fitness comparable to that of ptA_WT*IN* was the patient-derived full-length *pol* or *IN* only virus, expressing the rare *IN* Y143G + G163R mutation combination at 86.9 ± 7.6 and 102 ± 6.6%, respectively (*p* = 0.28 and 0.85). All other viruses expressing patient-derived full-length *pol* or *IN* only had significantly lower replicative fitness than ptA_WT*IN*, ranging from 12.9 ± 0.6 to 72.9 ± 3.4% (*p* ≤ 0.01). 

**Figure 6:**
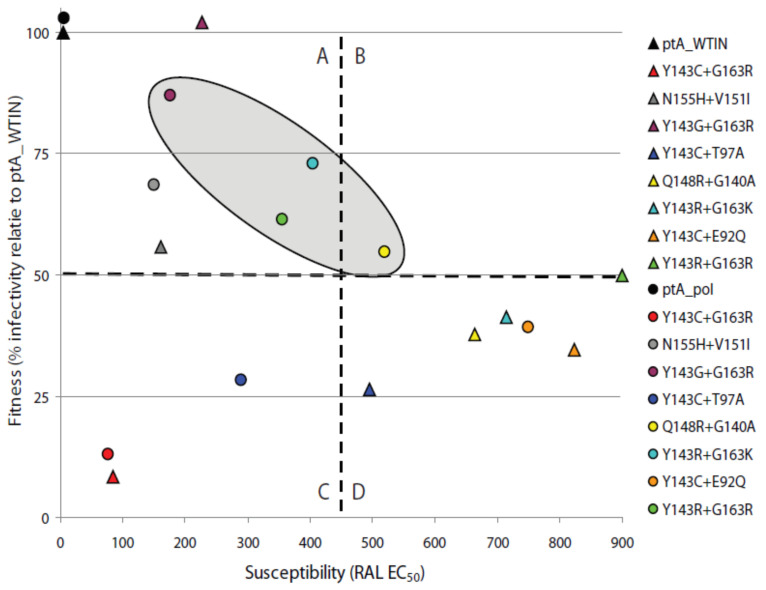
Relationship between the replicative fitness and RAL susceptibility exhibited by recombinant viruses from ptA. A graph plotting the relationship between replicative fitness and RAL susceptibility of recombinant viruses expressing either patient-derived *IN* gene only or full-length *pol* gene. The graph is equally divided into four hypothetical quadrants: (**A**) high replicative fitness (>50%) and low RAL resistance (<450 nM), (**B**) high replicative fitness (>50%) and RAL resistance (>450 nM), (**C**) low replicative fitness (<50%) and RAL resistance (>450 nM), (**D**) low replicative fitness (<50%) and high RAL resistance (<450 nM). Triangles represent viruses expressing patient-derived *IN* gene only; circles represent the respective viruses expressing full-length *pol* gene. The shaded oval represents viruses that dominated the viral population during RAL-containing salvage therapy at 4RAL, 5RAL and reRAL.

In general, the replicative fitness of viruses expressing full-length *pol* was greater than or comparable to that of viruses expressing the respective *IN* gene only. The viruses showing significantly increased replicative fitness upon expression of patient-derived full-length *pol* relative to *IN* gene only were those with the following RAL resistance mutation combinations: Q148R + G140A (*p* = 0.0023), Y143R + G163R (*p* = 0.024), Y143R + G163K (*p* ≤ 0.0001) and Y143C + G163R (*p* = 0.013). Furthermore, viruses that dominated the population during the two RAL treatment phases had both high levels of RAL resistance and replication fitness (Figure 6).

## 3. Discussion

InSTIs are a preferred third agent in NRTI-based regimen. Consequently, some patients on an InSTI-containing regimen will or have previously failed therapies containing PIs and RTIs, especially in low- and middle-income countries. Thus, it is important to understand the interaction and development of drug resistance mutations in the context of patient-derived full-length *pol* gene. In this study, we used a full-length HIV-1 *pol* gene SGS assay to demonstrate the genetic linkage of drug resistance mutations throughout the *pol* gene. We showed that drug resistance mutations in IN are linked to those in PR and RT, and that different combinations of InSTI resistance mutations can develop concurrently linked to the same PR and RT drug resistance mutations. This is consistent with another study showing the linkage of EVG resistance mutations in IN to drug resistance mutations in PR and RT [15]. 

Our data also revealed the simultaneous presence of mutations at all three major InSTI drug resistance positions (Q148, Y143 and N155) during treatment failure, albeit on different genomes, which is also consistent with previous findings [19,20,21,22,23]. In this study, the major InSTI resistance mutations were always linked to accessory mutations. This may be due to a longer period between initiation of RAL therapy and first sampling, which was at least 4 months. It is well established that major RAL resistance mutations can appear rapidly (sometimes within a month) after initiation of RAL treatment, with accessory mutations developing subsequently to compensate for fitness loss and/or to increase drug resistance [19,20,21,22,23,24,25,26,27,28,29]. The genetic linkage of major and accessory mutations suggests that the accessory mutations compensate for fitness loss and/or increase drug resistance of a viral variant through cis-acting mechanisms. This has been confirmed for the Q148H + G140S double mutant, in which it was demonstrated that the catalytic properties of IN were greatly impaired by the single mutants. However, the double mutant could fully restore the catalytic properties of IN only when the two mutations were present on the same IN polypeptide [28]. 

Our findings also shed light on the complexities of the intrapatient evolution of the Y143 resistance pathway, and the effects of accessory mutations linked to this pathway. Previous studies suggest that the accessory mutations linked to the Y143 mutations play a positive role in IN activity and/or RAL resistance [21,24,30]. In this study, we observed that different accessory mutations combined with a particular substitution at position Y143 differentially influenced the levels of susceptibility to RAL. For example, we observed the development of the Y143C resistance mutation, linked to three different accessory mutations (E92Q, T97A and G163R), differentially decreased RAL susceptibility from 19- to 183-fold, as well as viral fitness by 8 to 35% compared to wild-type virus. All three of the accessory mutations are found in the vicinity of the catalytic active site of *IN* [31]; therefore, it is envisioned that they could be affecting susceptibility and replicative fitness by directly influencing the structure of the active site.

The differential effect on RAL susceptibility and viral fitness also extended to different substitutions at primary resistance position 143 (R, C or G), linked to the same accessory mutation (G163R). This finding is contrary to another study which showed that the RAL susceptibilities of both the Y143R and Y143C mutants were similar [30]. However, in this study, the Y143 mutants were not linked to the G163R accessory mutation, which may partially explain the different observations. These data suggest that accessory mutations linked to Y143 mutations affect both the replicative fitness and InSTI susceptibility of the mutant viruses, and that a balance between the two could play a role in determining the development and evolution of resistance. Consistent with this observation, the Y143G + G163R mutant, that was present as a minority variant after RAL treatment was stopped, emerged as the dominant viral variant upon the re-initiation of RAL therapy. This variant exhibited a significantly higher RAL EC_50_ than the Y143C + G163R mutant, and had a significantly higher replicative fitness compared to the Y143R + G163R and Y143C + G163R mutants. The replicative fitness of Y143G + G163R was in fact the highest of all RAL resistant variants identified in this patient, and was comparable to that of wild-type virus. It is therefore likely that the subsequent outgrowth of this viral variant after the re-initiation of RAL therapy may be due to its higher replicative fitness compared to the other RAL resistant viruses present in the patient. The late and rare development of the Y143G substitution could be due to a high genetic barrier. The wild-type codon for position 143 in IN in this patient was TAC. One nucleotide change was required for the Y to C substitution (TAC to TGC), whilst two nucleotide changes were needed to generate the G substitution (TAC to GGC) or R substitution (TAC to CGC). The second change required for the Y to R substitution (T to C) is a transition which occurs at a higher frequency compared to the T to G transversion required for the Y to G substitution. Thus, although more advantageous to the virus in terms of fitness, the Y143G substitution is likely to occur less frequently compared to Y143R/C substitutions. This is supported by analysis of data available from the Stanford HIV Drug Resistance Database, which showed no instances of Y143G mutation in InSTI-experienced subtype B infected patients (Figure 1B). This also illustrates that continuous selective drug pressure during a failing regimen will force the virus to continue evolving towards a fitter resistant virus, that is then more likely to persist in the absence of drug pressure [32,33].

A limitation of the study is that the analysis, although in depth, is from one patient. Future studies will focus on investigating this phenomenon in a large group of patients, and include investigation of resistance to second generation InSTIs, such as DTG and use of full-length genome clones. Nonetheless, the data show that the coevolved *PR* and *RT* genes affect the susceptibility and replicative fitness of an *IN* gene harbouring InSTI resistance mutations by up to three-fold. This concurs with another study, that showed an effect on viral fitness and susceptibility to EFV and RAL for certain combinations of NNRTI and InSTI resistance mutations [34]. On the other hand, other studies have shown that mutations in PR and RT have little effect on the susceptibility to InSTIs, but can reduce viral replicative fitness of a resistant *IN* gene [35]. Different experimental approaches, such as the use of site-directed mutants compared to patient-derived fragments, or differences in the combination of resistance mutations and/or accessory mutations, could explain the contradictory outcomes. Therefore, further comprehensive studies coupling biological and biochemical investigations are required to elucidate the interactions between mutations in full-length HIV-1 *pol* gene and their effects on susceptibility and viral fitness, including that to the new InSTIs DTG, BIC and CAB. However, taken together, these data suggest that analysis of only part of the HIV-1 genome is probably not sufficient to gauge the true dynamics in the evolution and extent of drug resistance in the era of cART, as shown by recent studies linking InSTI resistance to env and cPPT regions [33,36,37]. The use of assays encompassing full-length *pol* gene or more of the viral genome may provide useful insights into drug resistance mechanisms, and help devise better treatment strategies as well as improve the prediction of the emergence of drug resistance and subsequent treatment failure.

## 4. Materials and Methods

### 4.1. Clinical Samples

The plasma samples used in this study were obtained from a patient attending the Mortimer Market Clinic, UCLH, who was infected with subtype B virus and initiated on RAL salvage therapy (600 mg daily) in September 2007. They continued RAL in combination with darunavir/ritonavir (DRV/r) and etravirine (ETR) until February 2008, when the patient experienced virological failure. The patient was then switched onto therapy containing tenofovir (TDF) and lamivudine (3TC), but experienced virological failure again 2 months later (April 2008). RAL treatment was then re-started in combination with TDF/emtricitabine (FTC), DRV/r and ETR in September 2008. Six samples were obtained, and these were: pre-RAL therapy (preRAL); 2, 4 and 5 months on RAL (2RAL, 4RAL and 5RAL, respectively); 4 months after RAL was stopped (4post); and 0.5 months after RAL was re-started (reRAL) [23]. Informed consent was obtained in the context of routine resistance testing as a part of clinical protocol.

### 4.2. Single-Genome Sequencing

A previously described, single genome sequencing assay [38] was modified by designing new antisense primers at the end of *IN*, and used to sequence full-length *pol* from sequential samples. cDNA synthesis and single genome PCR reactions were carried out as described, but using the antisense primer KVL069 (5′-TTCTTCCTGCCATAGGARATGCCTAAG-3′) [39] followed by a nested PCR reaction using either 5095- (5′-TAATCCTCATCCTGTCTACYTGCCACAC-3′), KVL084 (5′-TCCTGTATGCARACCCCAATATG-3′) [39] or 5222-deg (5′-TGTCTATAAAACCATCCTYTAGC-3′) antisense primers.

### 4.3. Single-Replication Cycle Drug Susceptibility Assay

To study phenotypic drug susceptibility, we used a previously described three plasmid-based retroviral vector system, utilising luciferase expression as a measure of infectivity [13,40]. The p8.9NSX *gag-pol* expression vector, which contains a unique *Apa*I restriction site in *gag* (upstream of the *PR* start codon) and an *EcoR*I restriction site in *vif* (downstream of the *IN* stop codon), was modified by introducing a unique *Cla*I restriction site at the beginning of *IN* (flanking amino acids 4/5), creating the p8.9NSXClaI+ vector. In parallel, mutagenesis was used to introduce the same *Cla*I and *EcoR*I sites in nine of the single genomes amplified from the patient in the single genome assay. The nine single genome variants were selected from different on- and off-RAL treatment time points, which included all the different RAL resistance mutation combinations observed in the patient, with these being: N155H + V151I, Q148R + G140A, Y143R + G163R, Y143R + G163K, Y143C + E92Q, Y143C + T97A, Y143C + G163R, Y143G + G163R as well as a wild-type *IN* from the pre-RAL time point (used as a control and designated ptA_WT*IN*).

In addition, the *Apa*I restriction site in *gag* and the *Cla*I restriction site at the beginning of *IN* were introduced into a single genome containing L10F, V32I, L33F, M46I, I47V, I54L, A71T, I84V, L89V and L90M in *PR*, and M41L, D67N, L74V, M184V, T215Y, K219E, L100I, K103N and N348I in *RT*. These resistance mutations were present in all single genomes chosen for the analysis. The unique restriction sites were then used for the subcloning of patient-derived *PR* + *RT* (*Apa*I and *Cla*I) or *IN* (*Cla*I and *EcoR*I) into p8.9NSXClaI+, to generate ptA*_PR* + *RT* and patient-derived *IN* only vectors, respectively (Figure 2). The generation of full-length *pol* vectors was achieved by subcloning patient-derived *IN* fragments into ptA*_PR* + *RT*, using the *Cla*I and *EcoR*I restriction sites. All mutagenesis was carried out using the QuikChange Lightning Multi Site-Directed Mutagenesis kit or QuikChange II XL Site-Directed Mutagenesis kit (Agilent Technologies), according to the manufacturer’s protocol. The presence of mutations was verified by sequencing.

The two other vectors used in this assay are the retroviral expression vector, pCSFLW, which encodes the firefly luciferase reporter gene, and pMDG, which encodes the vesicular stomatitis virus G protein. Viruses were generated by cotransfection of 293T cells as previously described, and then used to infect fresh 293T cells in the presence of serially diluted RAL or EVG [41,42]. The replication fitness of the virus was determined by p24 ELISA (Genscreen™ HIV-1 Ag Assay, Bio-Rad) and expressed as a percent of the ptA_WT*IN* control, as described previously [13].

### 4.4. Antiretroviral Drugs

RAL and EVG (repository references ARP980 and ARP991, respectively) were obtained from the National Institute for Biological Standards and Control.

### 4.5. Statistical Analyses

Differences in EC_50_ values and replicative fitness were calculated using the Student’s *t* test tool, available on www.graphpad.com (accessed on 10 June 2021). *p* values which were <0.05 were regarded as significant.

### 4.6. Nucleotide Sequence Accession Numbers

The single-genome sequences generated and used in this study have been submitted to GenBank and assigned the accession numbers MH663797-MH663975.

## Figures and Tables

**Table 1:** Clinical history of patient.

Patient	Months Before or After Initiation of RAL Therapy	Viral Load (Copies/mL)	CD4 Count (Cells/mm^3^)	Antiretroviral Treatment	Number of Single Genomes
A	−3 (preRAL)	59,000	150	LPVr, 3TC	16
	2 (2RAL)	140	230	DRVr, ETR, RAL	Na ^a^
	4 (4RAL)	39,000	280	DRVr, ETR, RAL	26
	5 (5RAL)	63,000	200	DRVr, ETR, RAL	23
	9 (4post)	77,300	140	TDF, 3TC	39
	14 (ReRAL)	1900	140	DRVr, TDF, FTC, ETR, RAL	13

^a^ na = no amplification, possibly due to low viral load.

## Data Availability

Sequence data generated by the study is available through NCBI GenBank and assigned the accession numbers MH663797-MH663975.

## References

[1] UNAIDS Joint United Nations Programme on HIV/AIDS (2020). UNAIDS Data 2020.

[2] Grobler J.A., Stillmock K., Hu B., Witmer M., Felock P., Espeseth A.S., Wolfe A., Egbertson M., Bourgeois M., Melamed J. (2002). Diketo acid inhibitor mechanism and HIV-1 integrase: Implications for metal binding in the active site of phosphotransferase enzymes. Proc. Natl. Acad. Sci. USA.

[3] Marchand C., Johnson A.A., Karki R.G., Pais G.C., Zhang X., Cowansage K., Patel T.A., Nicklaus M.C., Burke T.R., Pommier Y. (2003). Metal-dependent inhibition of HIV-1 integrase by beta-diketo acids and resistance of the soluble double-mutant (F185K/C280S). Mol. Pharmacol..

[4] Arribas J.R., Eron J. (2013). Advances in antiretroviral therapy. Curr. Opin. HIV AIDS.

[5] Steigbigel R.T., Cooper D.A., Teppler H., Eron J.J., Gatell J.M., Kumar P.N., Rockstroh J.K., Schechter M., Katlama C., Markowitz M. (2010). Long-term efficacy and safety of Raltegravir combined with optimized background therapy in treatment-experienced patients with drug-resistant HIV infection: Week 96 results of the BENCHMRK 1 and 2 Phase III trials. Clin. Infect. Dis..

[6] Churchill D., Waters L., Ahmed N., Angus B., Boffito M., Bower M., Dunn D., Edwards S., Emerson C., Fidler S. (2016). British HIV Association guidelines for the treatment of HIV-1-positive adults with antiretroviral therapy 2015. HIV Med..

[7] European AIDS Clinical Society Guidelines 2018. https://www.eacsociety.org/files/2018_guidelines-9.1-english.pdf.

[8] Mbisa J.L. (2013). Antiviral Resistance Testing. eLS. https://onlinelibrary.wiley.com/doi/abs/10.1002/9780470015902.a0024795.

[9] Avila-Rios S., Parkin N., Swanstrom R., Paredes R., Shafer R., Ji H., Kantor R. (2020). Next-Generation Sequencing for HIV Drug Resistance Testing: Laboratory, Clinical, and Implementation Considerations. Viruses.

[10] Hertogs K., de Bethune M.P., Miller V., Ivens T., Schel P., Van Cauwenberge A., Van Den Eynde C., Van Gerwen V., Azijn H., Van Houtte M. (1998). A rapid method for simultaneous detection of phenotypic resistance to inhibitors of protease and reverse transcriptase in recombinant human immunodeficiency virus type 1 isolates from patients treated with antiretroviral drugs. Antimicrob. Agents Chemother..

[11] Kellam P., Larder B.A. (1994). Recombinant virus assay: A rapid, phenotypic assay for assessment of drug susceptibility of human immunodeficiency virus type 1 isolates. Antimicrob. Agents Chemother..

[12] Petropoulos C.J., Parkin N.T., Limoli K.L., Lie Y.S., Wrin T., Huang W., Tian H., Smith D., Winslow G.A., Capon D.J. (2000). A novel phenotypic drug susceptibility assay for human immunodeficiency virus type 1. Antimicrob. Agents Chemother..

[13] Parry C.M., Kohli A., Boinett C.J., Towers G.J., McCormick A.L., Pillay D. (2009). Gag determinants of fitness and drug susceptibility in protease inhibitor-resistant human immunodeficiency virus type 1. J. Virol..

[14] Weber J., Vazquez A.C., Winner D., Rose J.D., Wylie D., Rhea A.M., Henry K., Pappas J., Wright A., Mohamed N. (2011). Novel method for simultaneous quantification of phenotypic resistance to maturation, protease, reverse transcriptase, and integrase HIV inhibitors based on 3'Gag(p2/p7/p1/p6)/PR/RT/INT-recombinant viruses: A useful tool in the multitarget era of antiretroviral therapy. Antimicrob. Agents Chemother..

[15] Winters M.A., Lloyd R.M., Shafer R.W., Kozal M.J., Miller M.D., Holodniy M. (2012). Development of elvitegravir resistance and linkage of integrase inhibitor mutations with protease and reverse transcriptase resistance mutations. PLoS ONE.

[16] Van Baelen K., Rondelez E., Van Eygen V., Arien K., Clynhens M., Van den Zegel P., Winters B., Stuyver L.J. (2009). A combined genotypic and phenotypic human immunodeficiency virus type 1 recombinant virus assay for the reverse transcriptase and integrase genes. J. Virol. Methods.

[17] Low A., Prada N., Topper M., Vaida F., Castor D., Mohri H., Hazuda D., Muesing M., Markowitz M. (2009). Natural polymorphisms of human immunodeficiency virus type 1 integrase and inherent susceptibilities to a panel of integrase inhibitors. Antimicrob. Agents Chemother..

[18] Garrido C., Villacian J., Zahonero N., Pattery T., Garcia F., Gutierrez F., Caballero E., Van Houtte M., Soriano V., de Mendoza C. (2012). Broad phenotypic cross-resistance to elvitegravir in HIV-infected patients failing on raltegravir-containing regimens. Antimicrob. Agents Chemother..

[19] Charpentier C., Karmochkine M., Laureillard D., Tisserand P., Belec L., Weiss L., Si-Mohamed A., Piketty C. (2008). Drug resistance profiles for the HIV integrase gene in patients failing raltegravir salvage therapy. HIV Med..

[20] Malet I., Delelis O., Soulie C., Wirden M., Tchertanov L., Mottaz P., Peytavin G., Katlama C., Mouscadet J.F., Calvez V. (2009). Quasispecies variant dynamics during emergence of resistance to raltegravir in HIV-1-infected patients. J. Antimicrob. Chemother..

[21] Reigadas S., Anies G., Masquelier B., Calmels C., Stuyver L.J., Parissi V., Fleury H., Andreola M.L. (2010). The HIV-1 integrase mutations Y143C/R are an alternative pathway for resistance to Raltegravir and impact the enzyme functions. PLoS ONE.

[22] Fransen S., Gupta S., Danovich R., Hazuda D., Miller M., Witmer M., Petropoulos C.J., Huang W. (2009). Loss of raltegravir susceptibility by human immunodeficiency virus type 1 is conferred via multiple nonoverlapping genetic pathways. J. Virol..

[23] Ferns R.B., Kirk S., Bennett J., Cook P.M., Williams I., Edwards S., Pillay D. (2009). The dynamics of appearance and disappearance of HIV-1 integrase mutations during and after withdrawal of raltegravir therapy. AIDS.

[24] Canducci F., Marinozzi M.C., Sampaolo M., Boeri E., Spagnuolo V., Gianotti N., Castagna A., Paolucci S., Baldanti F., Lazzarin A. (2010). Genotypic/phenotypic patterns of HIV-1 integrase resistance to raltegravir. J. Antimicrob. Chemother..

[25] Baldanti F., Paolucci S., Gulminetti R., Brandolini M., Barbarini G., Maserati R. (2010). Early emergence of raltegravir resistance mutations in patients receiving HAART salvage regimens. J. Med. Virol..

[26] Fun A., Van Baelen K., van Lelyveld S.F., Schipper P.J., Stuyver L.J., Wensing A.M., Nijhuis M. (2010). Mutation Q95K enhances N155H-mediated integrase inhibitor resistance and improves viral replication capacity. J. Antimicrob. Chemother..

[27] Delelis O., Malet I., Na L., Tchertanov L., Calvez V., Marcelin A.G., Subra F., Deprez E., Mouscadet J.F. (2009). The G140S mutation in HIV integrases from raltegravir-resistant patients rescues catalytic defect due to the resistance Q148H mutation. Nucleic Acids Res..

[28] Metifiot M., Maddali K., Naumova A., Zhang X., Marchand C., Pommier Y. (2010). Biochemical and pharmacological analyses of HIV-1 integrase flexible loop mutants resistant to raltegravir. Biochemistry.

[29] Nakahara K., Wakasa-Morimoto C., Kobayashi M., Miki S., Noshi T., Seki T., Kanamori-Koyama M., Kawauchi S., Suyama A., Fujishita T. (2009). Secondary mutations in viruses resistant to HIV-1 integrase inhibitors that restore viral infectivity and replication kinetics. Antivir. Res..

[30] Delelis O., Thierry S., Subra F., Simon F., Malet I., Alloui C., Sayon S., Calvez V., Deprez E., Marcelin A.G. (2010). Impact of Y143 HIV-1 integrase mutations on resistance to raltegravir in vitro and in vivo. Antimicrob. Agents Chemother..

[31] Mbisa J.L., Martin S.A., Cane P.A. (2011). Patterns of resistance development with integrase inhibitors in HIV. Infect. Drug Resist..

[32] Mbisa J.L., Gupta R.K., Kabamba D., Mulenga V., Kalumbi M., Chintu C., Parry C.M., Gibb D.M., Walker S.A., Cane P.A. (2011). The evolution of HIV-1 reverse transcriptase in route to acquisition of Q151M multi-drug resistance is complex and involves mutations in multiple domains. Retrovirology.

[33] Malet I., Delelis O., Nguyen T., Leducq V., Abdi B., Morand-Joubert L., Calvez V., Marcelin A.G. (2019). Variability of the HIV-1 3′ polypurine tract (3′PPT) region and implication in integrase inhibitor resistance. J. Antimicrob. Chemother..

[34] Hu Z., Kuritzkes D.R. (2014). Altered viral fitness and drug susceptibility in HIV-1 carrying mutations that confer resistance to nonnucleoside reverse transcriptase and integrase strand transfer inhibitors. J. Virol..

[35] Gupta S.F., Frantzell A., Chappey C., Petropoulos C., Huang W. Combinations of primary NNRTI- and integrase inhibitor-resistance mutations do not alter HIV-1 drug susceptibility but impair replication capacity. Proceedings of the 16th Conference on Retroviruses and Opportunistic Infections.

[36] Van Duyne R., Kuo L.S., Pham P., Fujii K., Freed E.O. (2019). Mutations in the HIV-1 envelope glycoprotein can broadly rescue blocks at multiple steps in the virus replication cycle. Proc. Natl. Acad. Sci. USA.

[37] Malet I., Subra F., Charpentier C., Collin G., Descamps D., Calvez V., Marcelin A.G., Delelis O. (2017). Mutations Located outside the Integrase Gene Can Confer Resistance to HIV-1 Integrase Strand Transfer Inhibitors. MBio.

[38] Palmer S., Kearney M., Maldarelli F., Halvas E.K., Bixby C.J., Bazmi H., Rock D., Falloon J., Davey R.T., Dewar R.L. (2005). Multiple, linked human immunodeficiency virus type 1 drug resistance mutations in treatment-experienced patients are missed by standard genotype analysis. J. Clin. Microbiol..

[39] Van Laethem K., Schrooten Y., Covens K., Dekeersmaeker N., De Munter P., Van Wijngaerden E., Van Ranst M., Vandamme A.M. (2008). A genotypic assay for the amplification and sequencing of integrase from diverse HIV-1 group M subtypes. J. Virol. Methods.

[40] Gupta R.K., Kohli A., McCormick A.L., Towers G.J., Pillay D., Parry C.M. (2010). Full-length HIV-1 Gag determines protease inhibitor susceptibility within in vitro assays. AIDS.

[41] Bainbridge J.W., Stephens C., Parsley K., Demaison C., Halfyard A., Thrasher A.J., Ali R.R. (2001). In vivo gene transfer to the mouse eye using an HIV-based lentiviral vector; efficient long-term transduction of corneal endothelium and retinal pigment epithelium. Gene Ther..

[42] Wright E., Temperton N.J., Marston D.A., McElhinney L.M., Fooks A.R., Weiss R.A. (2008). Investigating antibody neutralization of lyssaviruses using lentiviral pseudotypes: A cross-species comparison. J. Gen. Virol..

